# Declines in Spending Despite Positive Returns on Investment: Understanding Public Health's Wrong Pocket Problem

**DOI:** 10.3389/fpubh.2019.00159

**Published:** 2019-06-18

**Authors:** J. Mac McCullough

**Affiliations:** ^1^College of Health Solutions, Arizona State University, Phoenix, AZ, United States; ^2^Health Economist, Maricopa County Department of Public Health, Phoenix, AZ, United States

**Keywords:** public health spending, return on investment, wrong pocket problem, public health departments, public health economics

An axium attributed to Benjamin Franklin holds that *an ounce of prevention is worth a pound of cure*. While actual the exchange rate might differ, a recent study found that that every $1 invested in public health departments resulted in a remarkable $67 to $88 of benefits to society (1). Other studies have estimated smaller but still positive returns to public health spending (2, 3). In theory, health stakeholders, policymakers, taxpayers, and even private investors would flock to finance investments with such positive returns on investment. Yet public health spending in the U.S. has fallen as proportion of total health spending beginning around 2000 and in inflation-adjusted terms since the Great Recession (4). These declines have resulted in cuts to the public health workforce and to public health program portfolios (5, 6). What gives rise to such paradoxical findings and what mechanisms exist that may help steer public health spending toward an optimal level?

The core mission of public health is to promote and protect the health of people and the communities where they live, learn, work, and play (7). When a governmental public health agency succeeds in its mission, that agency may not be the direct beneficiary of that success. Instead the benefits accrue to other agencies. For example, when a measles outbreak investigation identifies an unvaccinated person, prevents their exposure to an infectious individual, and prevents their contracting the disease, many thousands of dollars of treatment costs are likely averted. While we would be right to celebrate this outcome from a public health perspective, a major beneficiary of this averted measles case would be the entity responsible for financing that person's health care—generally their health insurance provider (if insured), a charity care provider, or that individual's personal savings account. Thus, while the prevention work was financed by a governmental public health agency, the financial savings accrue to another entity entirely. The same principle applies to many other public health programs. In some cases, this challenge is exacerbated by the fact that the benefits accrue many years or decades into the future or are not tracable to any one individual.

This challenge is known as the “wrong pocket” problem. A wrong pocket problem can arise when one entity makes an investment in or bears costs for an initiative that, if successful, will generate benefits for a different entity. In other words, money comes out of one “pocket” (i.e., agency or budget area) and goes into a separate “pocket.”

Consider the case when public health is successful in its mission—say when a communicable disease outbreak is stemmed, a case of chronic disease prevented, or a risk factor mitigated. In each of these cases, public health has provided a clear benefit to the community in terms of improved health outcomes and or health status. Yet the public health agency itself is unlikely to reap any financial gains from this outcome. While it is true that financial gains are generally not the main goal of public health organizations, it is critical to note that when public health succeeds, there often times are entities that do realize financial gains. For example, private health insurers, Medicare, state Medicaid programs and others may enjoy reduced spending on health care services stemming from improvements in public health outcomes. Other potentially relevant beneficiaries include the full gamut of public agencies and social services providers that depend in part on the health of the community: housing agencies, schools, courts and jails, police and fire departments. State and federal tax agencies such as the IRS could benefit financially if persons are healthier and better able to learn, work, and live in their communities. Misalignment of expenditures and revenues means that financial savings that accrue as a result of successful public health programs are likely to be reflected on the budgets of other entities.

A major implication of the wrong pocket problem is that it can skew our ability to make good decisions about how much we spend on public health. When stakeholders are not fully aware of the costs or do not fully reap the benefits of that spending, we would not expect traditional supply and demand market forces to yield optimal spending allocations. In theory the beneficiaries of public health successes (e.g., private insurers, employers, etc.) would want to see successful public health programs funded up to or even past optimal levels. After all any additional spending on such programs could yield direct benefits to them without them having to pay the full direct costs of the program. However, the beneficiaries of public health's spending have little incentive to unilaterally invest in public health because they still stand to benefit regardless of whether or not they decide to invest their own resources. In economic parlance, these entities are incentivized to be freeriders, leading to a tragedy of the commons. Public health agencies, on the other hand, are left having to justify annual budget requests with limited ability to reap the savings from their programs' impacts.

While the public agencies on opposite sides of the wrong pocket problem may be supported by a similar set of taxpayers, building the evidence-base and political will to invest in these public health agencies at optimal levels can be challenging to build and sustain. For example if a public health program is anticipated to result in fewer high-school dropouts, data from state or local education agencies may not be easily obtainable, especially at granular levels. Likewise health care cost savings data may be challenging to obtain. Moreover, even if data are available, why would a city councilperson vote to spend (let alone raise) tax dollars for a public health program when the benefits flowing from that program flow to schools—which are funded largely by county governments—or health insurance plans—which are funded largely by federal and state governments and employers?

## Potential Policy Solutions for Tackling the Wrong Pocket Problem

Addressing public health's wrong pocket problem will require mechanisms through which costs and benefits are better aligned. In practice this involves incentivizing beneficiaries to share in the costs of the programs they benefit from. There may not be a magic bullet that solves the wrong pocket problem in every community for every public health program, but several overarching funding strategies may help to inform stakeholders and spur progress toward a more economically efficient funding approach to promoting and protecting the public's health.

Both social impact bonds (8) and pay for success (9) have been used to fund programs subject to the wrong pocket problem. Early results suggest some potential for these initiatives to improve health outcomes and sometimes yield positive returns for investors (10). Yet social impact bonds are unlikely to be a panacea for reaching optimal funding levels for the entire spectrum of public health services (11), especially if the outcomes of a public health program occur years or decades into the future or are not easily tracable back to specific populations. and there are concerns over their use as an approach to funding public health programs (12).

Value-based payment approaches for health care may help bolster financial incentives for providers invest in public health programs that improve health (13). Yet the current generation of payment models tend to fall short of the paradigm-shifting approaches that may be needed to eliminate wrong pocket problems and fund public health at economically-optimal levels (14).

Reforming governmental agencies may also offer promise. If the core of the issue is that siloed government agencies' pockets don't align, one clear solution is to de-silo so that pockets do align.

Finally, if the crux of the wrong pocket problem are shortages of political will (which may be circumvented in part through the strategies above) and available evidence, this suggests a clear opportunity for additional evidence to have a direct impact on the ability for public health interventions to be funded at economically efficient levels. Evidence that estimates stakeholder-specific returns over policy-relevant timelines would be essential. Building evidence that can be directed toward specific audiences regarding the benefits that would accrue to a specific stakeholder such as a county's health budget or total budget, a state Medicaid agency, or to CMS programs may further enhance the impact of public health ROI studies.

A growing body of literature is showing that public health spending can yield positive economic returns (to say nothing of its health and human impacts). Yet the wrong pocket problem threatens to dampen the impact of that evidence. Reforms to how programs are funded, how governments are structured, and how evidence is generated may help to ensure that levels of spending that are commensurate with public health's true return to society.

## Author Contributions

The author confirms being the sole contributor of this work and has approved it for publication.

### Conflict of Interest Statement

The author declares that the research was conducted in the absence of any commercial or financial relationships that could be construed as a potential conflict of interest.
